# Insights from analyses of low complexity regions with canonical methods for protein sequence comparison

**DOI:** 10.1093/bib/bbac299

**Published:** 2022-08-01

**Authors:** Patryk Jarnot, Joanna Ziemska-Legiecka, Marcin Grynberg, Aleksandra Gruca

**Affiliations:** Department of Computer Networks and Systems, Silesian University of Technology, Akademicka 2A, 44-100, Gliwice, Poland; Institute of Biochemistry and Biophysics, Polish Academy of Sciences, Pawinskiego 5A, 02-106, Warsaw, Poland; Institute of Biochemistry and Biophysics, Polish Academy of Sciences, Pawinskiego 5A, 02-106, Warsaw, Poland; Department of Computer Networks and Systems, Silesian University of Technology, Akademicka 2A, 44-100, Gliwice, Poland

**Keywords:** comparison methods, low complexity regions, protein sequence similarity

## Abstract

Low complexity regions are fragments of protein sequences composed of only a few types of amino acids. These regions frequently occur in proteins and can play an important role in their functions. However, scientists are mainly focused on regions characterized by high diversity of amino acid composition. Similarity between regions of protein sequences frequently reflect functional similarity between them. In this article, we discuss strengths and weaknesses of the similarity analysis of low complexity regions using BLAST, HHblits and CD-HIT. These methods are considered to be the gold standard in protein similarity analysis and were designed for comparison of high complexity regions. However, we lack specialized methods that could be used to compare the similarity of low complexity regions. Therefore, we investigated the existing methods in order to understand how they can be applied to compare such regions. Our results are supported by exploratory study, discussion of amino acid composition and biological roles of selected examples. We show that existing methods need improvements to efficiently search for similar low complexity regions. We suggest features that have to be re-designed specifically for comparing low complexity regions: scoring matrix, multiple sequence alignment, e-value, local alignment and clustering based on a set of representative sequences. Results of this analysis can either be used to improve existing methods or to create new methods for the similarity analysis of low complexity regions.

## Introduction

Protein sequences are composed of amino acid fragments of varying diversity. Fragments with low diversity in the amino acid composition are called low complexity regions (LCRs). Due to their frequent occurrence and capacity to expand through replication slippage, they can easily increase protein sequence space and contribute to novel protein functions. They are known to play a key role in protein functions and may be relevant to protein structure [1]. For example, prion-like LCRs are key regulators of protein solubility and folding [2]. Cytoplasmic human Gle1 is hyperphosphorylated in a low-complexity domain in response to stress [3]. A known LCR motif RGG/RG is generally required for RNA binding and phase separation [4]. LCRs may also form labile cross-$\beta $ polymers and hydrogel droplets [5].

Methods and algorithms for searching for similarities among protein sequences have always been important tools in biology that allowed researchers to predict protein functions from the sequence data alone. Many approaches are known from the literature that are suitable for searching for similar protein sequences. However, these methods are based on statistical models that are optimized to compare high complexity fragments. Due to that, for many years, protein regions that are characterized by low complexity of amino acids had been ignored and excluded from such type of analysis.

Recently, the research community became more interested in the so-called Dark Proteome that is mostly composed of intrinsically disordered proteins or proteins that contain intrinsically disordered regions [6–8]. Therefore, nowadays, it is crucial to revisit state-of-the art methods of protein sequence comparison in order to understand if and how they can be applied to analyse similarity of LCRs.

The community has already made some efforts to develop tools that are capable of automatically assigning functional roles of LCRs. One such example is the web server LCR hound [9] which identifies Uniprot-annotated prokaryotic LCR sequences that have the closest amino acid or di-amino acid content in comparison to the query LCR sequence and based on this predicts a potential role of the query LCR. However, the prediction algorithm is using an amino acid content rather than sequence similarity, and therefore, we still lack of state-of-the-art methods for searching for similar LCRs in protein sequences.

Here, we present a study that compares the performance of three state-of-the art methods for searching for similarities among protein sequences: BLAST [10], HHblits [11] and CD-HIT [12]. By analysing how these methods perform in a task of searching for similar LCRs, we try to answer the following question: can these methods be applied to analyze LCRs or maybe new methods have to be invented? According to our best knowledge, scientists lack methods designed specifically for analyses of similarities among low complexity fragments of protein sequences. The aim of this study is also to raise awareness that the statistical models created for High Complexity Regions (HCRs) of proteins cannot be applied directly for a task of low complexity sequences comparison.

## Methods

**Figure 1:**
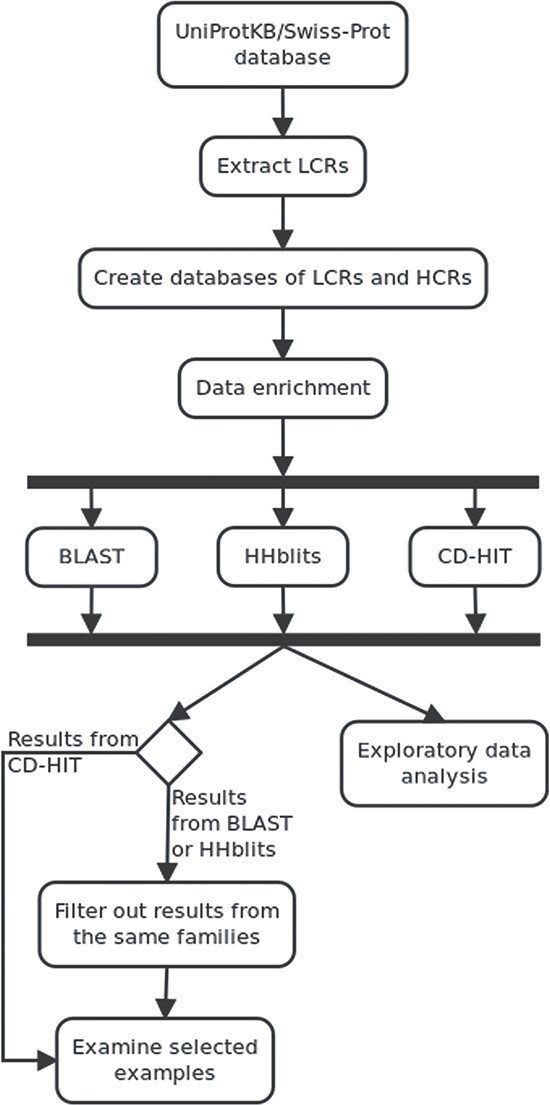
From the UniProtKB/Swiss-Prot database, we extracted LCRs and created two distinct datasets (HCRs and LCRs) enriched with family annotation that we analysed with BLAST, HHblits and CD-HIT. Then, we compared obtained similarity results and examined selected examples.

The workflow of our experimental approach is shown in Figure 1. In the analysis, we need high-quality annotated data, and therefore, we used the UniProtKB/Swiss-Prot database (version: April of 2020) [13]. We identified LCRs and we divided all sequences into LCR and HCR parts. If a sequence had several LCRs, it was split into these different LCRs and the remaining HCR part of the sequence. Then, we created two datasets with sequences collected in the previous step for both LCRs and HCRs. At this point, the dataset contained amino acid sequences with simple annotations (UniProt AC and a name of a protein it belongs to). In the next step, we added information about protein families and analysed this set of sequences with BLAST, HHblits and CD-HIT tools. Then, we evaluated these methods using exploratory analysis, by looking at amino acid composition and biological role of selected results based on UniProtKB/Swiss-Prot functional annotations. To select interesting cases from BLAST and HHblits results, we filtered out results from the same families. We performed the entire process several times adjusting the parameters for each method to achieve the best results. To select similar sequences, we used e-value threshold equal to 0.0001 for BLAST and HHblits. The source code for the entire workflow is available for download (https://doi.org/10.5281/zenodo.6759535).

### LCR extraction

The definition of LCR in a protein sequence is not well specified. General agreement is that LCRs in proteins should have an excess of one or a few types of amino acid residues, but still, there is no consensus which metric is most appropriate. LCRs can be composed of homopolymers, short tandem repeats and irregular regions with low entropy. The scientific literature provides different terminology and definitions of these regions that are typically based on the sequence composition and periodicity [14]. CAST [15] and fLPS [16] are the methods capable of detecting compositionally biased regions (CBRs). While the usage of the terms LCR and CBR has been interchangeable in many contexts, use of one term or the other depends on the focus of the method used for their detection, i.e. sequence variability (LCRs) or amino acid composition (CBRs), respectively [14]. Another type of LCRs are short tandem repeats which are characterized by amino acid periodicity (i.e. repetitiveness). The example of methods that are designed specifically to discover short tandem repeats is XSTREAM [17] and T-REKS [18]. SIMPLE [19] is another method that allows to detect so-called cryptic repeats [19] which are regions of proteins containing overrepresentations of short amino acid repeats.

Here, we consider LCRs as sequences characterised by low entropy in amino acids composition. This assumption is based on the definition provided by authors of the SEG method which is one of the most popular tools for searching for LCRs [20].

**Figure 2:**
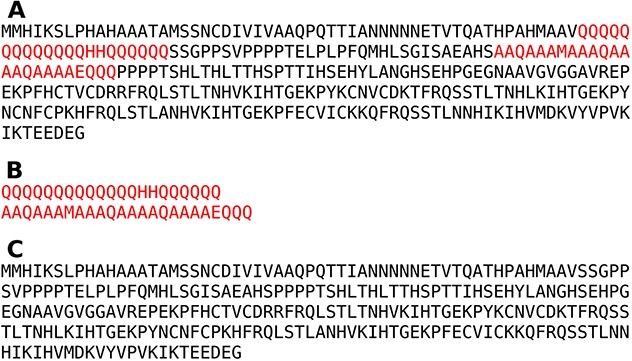
Division of the exemplary sequence (UniProtID: P39413) into a HCRs and LCRs. Panel (**A**) shows the whole sequence with identified LCRs highlighted in red. Panel (**B**) shows extracted LCRs and panel (**C**) presents all remaining non-LCR residues combined into an HCR part.

To identify LCRs, we used SEG strict parameters ($K$1: 1.5, $K$2: 1.8, window: 15) which have been successfully employed in LCR analyses. Strict parameters of SEG ensure that identified regions are strongly compositionally biased while also allowing for a low amino acid diversity [21, 22]. The first dataset is for LCRs and the second one for HCRs. Each sequence that includes one or more LCRs is split into its corresponding LCRs, while the remaining residues are joined creating the HCR part of the sequence as presented in Figure 2. As a result, we found 26 333 LCRs in 16 418 proteins from which we created two distinct datasets. The number of HCRs is equal to the number of proteins in the database that is 562 252.

### Creation of databases for LCRs and HCRs

The main purpose of our research is to compare canonical sequence searching/clustering methods for LCRs; however, we decided to compare HCRs as a control experiment that proves correctness of our workflow.

### Data enrichment

In the first step, we enriched protein sequences with information about their protein families based on UniprotKB/Swiss-Prot annotations. We need this information to exclude from the analysis similar sequences that are derived from the same family. The rationale behind excluding these sequences from direct comparison was because we expected a high level of similarity of protein sequences within the family. However, in our analyses, we wanted to focus on non-obvious cases where HCRs from two proteins are different but LCRs are similar.

### Parameters of the methods

This section presents in details the parameters used for analysis and adjustment of their values for analysis of low complexity parts of the sequences as by default, the methods’ parameters are optimised for high complexity parts (HCPs) of sequences.

#### BLAST

BLAST is the first method we used to search for similar sequences. It uses Smith–Waterman algorithm to calculate local alignment based on a query and database sequences [10]. We converted fasta formatted datasets to BLAST-specific format.

Below, we enumerate modified parameters. We set e-value to 0.0001 and *max_target_seqs* to maximal possible value (1 073 741 798). Additionally, to improve searching for LCRs using available BLAST options, we changed *task* and *comp_based_stats* parameters. A study of selected BLAST parameter settings that can be applied for LCRs analysis can be found in [23]. *Task* option is responsible for the default parameter set adjusted for specific types of sequences. Possible options are: *blastp*, *blastp-fast* and *blastp-short*. *Blastp-short* makes the following modifications to the default options: sets scoring matrix to PAM30, sets gap opening cost to 9, sets gap extension cost to 1, sets word size to 2, clears filter options and changes e-value (which is not applicable in our case because we explicitly set it). We left the scoring matrix parameter unchanged with its default value of PAM30 since it is recommended for short sequences [24]. *Comp_based_stats* option is responsible for composition-based statistics. This option changes the scoring matrix by recalculating score values of frequently occurring amino acids in the query sequence, which is mainly caused by LCRs. It simply decreases the significance of LCRs while searching [25], and therefore, we turned it off.

#### HHblits

HHblits is able to search for distantly related proteins that share a common ancestor. It is a part of the HH-Suite package and it uses Hidden Markov Model profiles to find similar sequences using HMM-HMM comparison [11]. Therefore, the query sequence is converted into an HMM profile. Database also stores HMM profiles which are condensed forms of multiple sequence alignments (MSAs) and represent protein sequences. In order to search for similar sequences, HHblits requires to create a database of profiles of Hidden Markov Models. We used uniclust-pipeline to create them for both LCRs and HCRs [26].

Uniclust-pipeline uses MMseqs to cluster similar sequences and to create their Hidden Markov Model profiles [26, 27]. We created two distinct datasets for both HCRs and LCRs. For HCRs, we used standard workflow, and for LCRs, we slightly modified it. To analyse LCRs with MMseqs tool, we changed two parameters. The first parameter is *mask* which is responsible for choosing a masking strategy and the second is *comp-bias-corr* which changes correction for locally biased composition of amino acids. While analysing LCRs, it is recommended to turn off both of these parameters by setting their values to 0. We also removed the *max-seqs* parameter as it is deprecated and not available in the newest version of MMseqs. The result of running uniclust-pipeline is several databases created with different identity thresholds: 10, 20 and 30%. From these results, we selected Uniboost30, a database with highest sequence identity that is equal to 30%. However, the threshold is still low, and therefore, the results obtained using this dataset contain more distant similarities.

We performed the analyses of both HCRs and LCRs using the same e-value as in the case of BLAST (0.0001). For LCR analysis, we modified the following parameters: *id*, *diff*, *norealign*, *sc* and *noprefilt*. These parameters control a number of results that are relevant to query sequences and their detailed analysis is provided in Supplementary material. *Id* parameter changes maximal pairwise sequence identity. We set this option to 100 (default is 90) because there are LCR families that are highly similar in their amino acid patterns. By default, HHblits selects the most diverse set of sequences, but here, we wanted to analyse the most similar ones, and therefore, we turned this setting off (*diff* parameter). Setting the *norealign* parameter disables MAC algorithm which significantly increases the number of matches [28]. *Sc* option changes the method which is used to calculate amino acid score. We examined all available parameters, and based on the empirical analyses, we chose the value of 0 which uses background frequencies. Default value for this parameter is optimized for compositional bias correction [29]. *noprefilt* parameter was introduced to speed up searches by filtering out cases that are ‘obviously’ not similar. However, we noticed that this parameter is filtering too many similar LCRs, so finally we disabled prefiltering.

**Figure 3:**
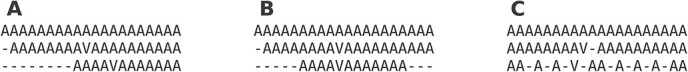
Possible ways of creating MSAs for LCRs collected from Q9V727, D3ZKD3 and Q5BGE2, respectively, that can generate different HMM profiles. These alignments were obtained using three different methods for MSA which are (**A**) MUSCLE, (**B**) Kalign and (**C**) Clustal Omega.

HHblits database contains profiles of HMMs which are a condensed form of MSA. Here, we notice an issue related to the MSA creation. In Figure 3, we can see three protein alignments of three different subsequences aligned by three different tools: MUSCLE [30], Kalign [31] and Clustal Omega [32]. All of these sequences are homopolymers of alanine, but two of them have a mutation into valine. The problem is that MSA may be created in several ways. For example, in Figure 3A, the third sequence was aligned to others by inserting gaps at the beginning of the sequence, while in Figure 3B, valine is aligned properly. Figure 3C presents the actual alignment based on Clustal Omega pipeline to create profiles of HMMs for HHblits. Different ways to obtain MSA results in different profiles HMM which influences searching outcome, because a particular position will be scored differently for given amino acid.

#### CD-HIT

The third method we analysed in this work is CD-HIT which uses a greedy incremental algorithm. In a nutshell, first all sequences are sorted by length in descendent order. Then, the longest sequence creates the first cluster. The sequence that creates a cluster is called a representative and all the following sequences are being aligned to it to determine whether they should be included into this cluster or not. If the similarity score of a particular aligned sequence is higher than a threshold, the sequence is added to the corresponding cluster. Otherwise, the sequence creates a new cluster and becomes its representative [33]. To speed up comparison time, short-word filtering and statistically based filtering were introduced [34]. We left default parameters unchanged for HCRs and we changed the minimal accepted length of sequences to its lowest value for LCRs, which is 4, since these regions are frequently short. We provide detailed analysis of this parameter in Supplementary material.

### Results analysis

We performed exploratory data analysis by investigating: (i) how the results from the selected methods overlap with each other, (ii) what is the average sequence similarity and (iii) what is the number of alignments for a given length? Finally, we present selected cases to show how BLAST, HHblits and CD-HIT analyse LCRs. We also analysed amino acid composition and biological roles of selected examples. To find more interesting cases, we filtered out results (hits) that include sequences from the same families for BLAST and HHblits. We assumed that minimal length of LCR is 6 amino acids.

To investigate biological features of the selected proteins, we performed a three layer functional analysis composed of the following stages: (i) We scanned popular DNA/protein databases (UniProt, RefSeq, STRING, Ensembl) [13, 35–37], (ii) Next we focused on the Pfam domain database [38] and (iii) the last stage was reading articles that mentioned specific proteins/protein families and their functional analyses.

## Results

### Quantitative results

**Figure 4:**
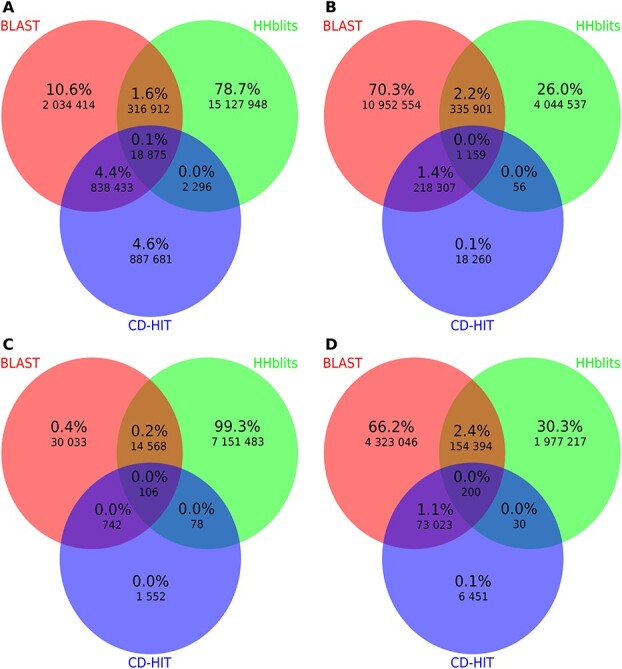
Venn diagrams present overlaps of the similar sequence pairs obtained by each of the methods. The only significant overlap is between BLAST and CD-HIT results. In the case of LCRs, CD-HIT results are subset of BLAST what is different for HCRs, where less than a half of results are common with BLAST. Other overlaps are slight, suggesting that the methods have different purposes. Panel (**A**) presents results for all HCRs, while panel (**C**) presents results for HCRs without pairs that belong to the same family. Panels (**B, D**) present the corresponding results for LCRs. Numbers in diagrams show the number of similar pairs of fragments found by specific method(s). Percentage determines how many pairs belong to the area versus all.

To quantitatively compare the results from the three methods, we created Venn diagrams (Figure 4). In addition, we used the HCR results as a reference for the comparison. BLAST and HHblits search for similar sequences; therefore, we have created similar pairs by combining a query sequence with each hit found by a particular method. CD-HIT is a tool for clustering highly homologous sequences [33]. We created pairs of similar sequences for CD-HIT by combining all possible pairs of sequences in clusters. Table 1 shows the number of similar pairs found by each method, while Figure 4 presents an overlap among them.

**Table 1:** In case of HCRs, HHblits found the highest number of similarities, while for LCRs, it was BLAST. CD-HIT reported low similarity between LCRs. We combined all similar pairs found by each method and calculated percentages which sum up to 100% in each of the columns

	HCR	LCR	HCRs without same families in pairs	LCRs without same families in pairs
BLAST	3,205,592 (15.83%)	11,507,921 (71.58%)	46,413 (0.65%)	4,550,663 (67.56%)
HHblits	15,296,119 (75.55%)	4,331,254 (26.94%)	7,096,205 (99.32%)	2,105,748 (31.26%)
CD-HIT	1,745,171 (8.62%)	237,782 (1.48%)	2,477 (0.03%)	79,705 (1.18%)

As shown in Table 1, the highest number of similar pairs for HCR results is found by HHblits (75.55% of all HCR pairs). If we remove pairs where both proteins belong to the same families, then HHblits results rises to 99.32% of all similar pairs found by all the methods. This was expected because HHblits is the most sensitive of the selected tools. On the other hand, in the case of LCRs, the highest number of similarities were found using BLAST which is over 71%, while HHblits results cover less than 27%. If we remove pairs that come from the same families in HCR parts for BLAST, then we remove almost 99% of similar pairs. In the case of LCRs, this number is about 60%. From all analysed methods, the lowest number of similar pairs was found by CD-HIT. In case of HCRs, it was 8.62% of all pairs, while for LCRs, it was only 1.48%. This may be due to its design and application as CD-HIT is mostly used to find sequence redundancy in datasets.

In Figure 4, we can observe that results for BLAST and HHblits are rather diverged in all cases. Only 1.6% of all HCR sequence pairs were found by both BLAST and HHblits and 2.2% in the case of LCRs. The differences between BLAST and HHblits results can be explained by the fact that BLAST finds closely related sequences, because it simply reports alignments with the lowest e-value. On the other hand, HHblits uses precalculated uniboost dataset that is optimized to boost diversity in sequences, and therefore, it finds alignments with more distant relationship. The intersection of CD-HIT and HHblits also has a small number of similar pairs. This observation is expected, because HHblits searches for distinct similarities while CD-HIT searches for close similarities or even can be used to detect redundancy in databases. Intersection of BLAST and CD-HIT shows that for HCRs, almost half of the results from CD-HIT overlaps with BLAST ( 49%). In the case of LCRs, most of the results are common with BLAST ( 92%). Intersection of all of the methods is poor for both HCRs and LCRs which may suggest that each of the selected methods covers different types of similarities. Remarkably, removing similar pairs that belong to the same families have a huge impact on HCR results, while LCR results are less affected.

For BLAST and HHblits results, we sorted alignments by e-value and divided them into 10 groups where each group contains about 10% of sequences. Therefore, the group size of BLAST is approximately 888 thousand pairs each and the group size of HHblits is approximately 222 thousand pairs. Within each group, we compared lengths of alignments to their similarity and the number of alignments. The similarity in an alignment between two sequences is denoted by percentage of similar amino acids where similar amino acids are these which have positive score in a scoring matrix.

**Figure 5:**
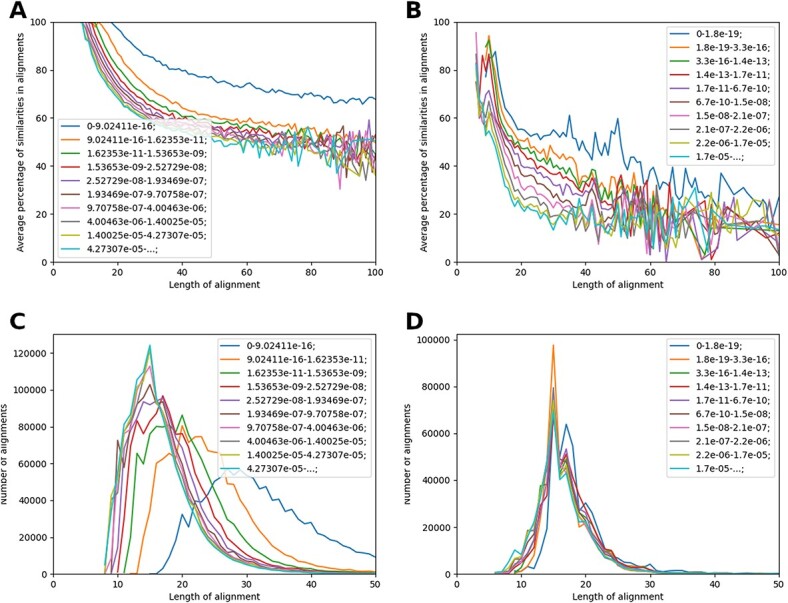
BLAST’s e-value is length and similarity-dependent, while HHblits e-value is only similarity dependent. We sorted results by e-value and divided them into 10 groups of similar size. (**A, C**) show the distribution of alignments by length. (**B, D**) illustrate how alignments of a given length are averagely similar.

For BLAST, the results for three best percentiles (the lowest e-value) differ from the results for other percentiles (Figure 5A). It suggests that for BLAST, e-value is length-dependent, and indeed, we can notice that length of alignments increases with decreasing e-value. Same situation, at least in the range from 0 to 100 amino acids, is visible in Figure 5B which shows average similarity of alignments for a given length. Along with increasing e-value, the average similarity in the percentiles tends to overlap. We can observe that alignments with lower e-value are longer and more similar than alignments with higher e-value, which is also reflected in higher score of alignments. Interestingly, HHblits results are different. In Figure 5C, e-value groups overlap which suggests that e-value is length-independent. In Figure 5D, we can observe that all groups are clearly divergent for alignments below 40, which is most of the results. Therefore, e-value in the HHblits results describes rather similarities between sequences than their length. It also indicates that alignments longer than 18aa are below 60% of similarity. Based on this results, we can notice that BLAST alignments are longer and more similar than HHblits alignments.

Corresponding figure for HCRs (Figure S1, see Supplementary Data available online at http://bib.oxfordjournals.org/) and comparison of lengths of alignments in different e-value percentiles for LCRs and HCRs are provided in Supplementary materials.

### Qualitative results

In this section, we discuss and analyse the most interesting cases (sequence alignments) obtained with the different methods. Here, we show data that indicate the following features: (i) some of the methods’ features are more appropriate for HCRs than LCRs and (ii) similarity of LCR sequences may but does not have to indicate a similar function of these sequences.

#### BLAST

For BLAST, we selected 5 representative alignments with their e-values that illustrate different problematic cases. At least, one of the proteins from alignments belongs to the krueppel C2H2-type zinc-finger protein family. Selected alignments are presented in Table 2

**Table 2:** BLAST can give ambiguous results; longer homopolymer of serine has higher e-value than shorter homopolymer of glutamine even though both homopolymers are aligned without penalty. Table shows perfect alignments collected from BLAST. It compares proteins from krueppel C2H2-type zinc-finger protein family (upper sequences) with proteins from other families (bottom sequences)

#	Protein name	Alignments	e-value
1	Zinc finger protein 865 (P0CJ78)	SSSSSSSSSSSSSSSSSSSSSSSSS (92 - 116)	1,10E-17
	midline	SSSSSSSSSSSSSSSSSSSSSSSSS	
	ADP-ribosylation factor-like protein 6-interacting protein 4 (Q66PJ3)	SSSSSSSSSSSSSSSSSSSSSSSSS (257 - 281)	
2	Zinc finger protein rotund (Q9VI93)	QQQQQQQQQQQQQQQQQQQQQQQ (739 - 761)	1,40E-23
	midline	QQQQQQQQQQQQQQQQQQQQQQQ	
	Ataxin-2 (Q99700)	QQQQQQQQQQQQQQQQQQQQQQQ (165 - 187)	
3	Zinc finger homeobox protein 4 (Q86UP3)	PPPPPPPPPPPP (3112 - 3123)	3,61E-10
	midline	PPPPPPPPPPPP	
	Inactive histone-lysine N-methyltransferase 2E (Q3UG20)	PPPPPPPPPPPP (1719 - 1730)	
4	Zinc finger homeobox protein 4 (Q86UP3)	PPPPPPPPPPPP (3112 - 3123)	1,84E-09
	midline	PPPPPPPPPPPP	
	Translation initiation factor IF-2 (Q2G5E7)	AAPTAAPAAAATPAPTPVAPPPPPPPPPPPP (53 - 83)	
5	Zinc finger homeobox protein 4 (Q86UP3)	PPPPPPPPPPPP (3112 - 3123)	7,75E-10
	midline	PPPPPPPPPPPP	
	Glyceraldehyde-3-phosphate dehydrogenase, testis-specific (Q64467)	PPPPPPPPPPPPPPPPPPPP (83 - 99)	

The first alignment presents homopolymers of serine that consist of 25 amino acids with e-value equal to $1,10E-17$ (Table 2). The second alignment contains homopolymers of glutamine that consists of 23 amino acids. It is two amino acid shorter than the first alignment but has a lower e-value ($1,40E-23$). This situation is caused by match score assigned to serine and glutamine in the PAM matrix [39]. Matches in this matrix have different scores; therefore, two homopolymers of different amino acids may have different scores.

The third, the fourth and the fifth examples show other issues that we can observe if we use BLAST to analyse similarities between LCRs. All of these alignments are 12 aa long. The third and the fifth alignments consist of homopolymers of proline. The third one is a perfect match over the total length of LCRs. On the other hand, the fifth alignment consists of two LCRs with different lengths resulting in an alignment with a shorter LCR length. As a result, both alignments have similar lengths and scores. However, the length of homopolymer in an alignment may be crucial for its function [40]. Therefore, the fourth alignment is much better than the fifth (because both homopolymers have the same length) but have a worse e-value than the fifth alignment. N-terminal of the bottom sequence in the 4th example is ‘AAPTAAPAAAATPAPTPVA’ which is an imperfection of the SEG algorithm that occurs because SEG is not able to distinguish LCRs in the first part of the subsequence from the second part that is a homopolymer of proline.

We used this opportunity to analyze biological properties of the LCR pairs presented in Table 2. Remarkably, the first pair represents a polyserine stretch (Table 2). The zinc finger 865 protein is a putative transcription factor composed mainly of zinc finger motifs and an undefined low complexity N end region. Zinc fingers bind to DNA, whereas the latter fragment is usually responsible for activation or repression of transcription [41]. Serine-rich regions are usually modified and most probably function as modulators of protein binding [42]. ADP-ribosylation factor-like protein 6-interacting protein 4 (ARL6IP4) is most probably involved in splicing in nuclear speckles; however, its exact role is unknown [43, 44]. The second pair represents human ataxin-2 and fly’s rotund proteins. Ataxin-2 polyQ region seems to be responsible for dimerization/aggregationr[45–47]. Since the function of Drosophila rotund’s polyQ is unknown, the parallel notion of dimerization is attractive in the context of developmental pathway in which rotund takes part [48] [49].

Moreover, a rare function can be contributed to the protein from three last examples. The polyproline stretch of ZFHX4, the zinc finger homeobox protein 4, serves as an assembly element for the human butyrylcholinesterase into a tetramer [50–52]. We could not find any other functions ascribed to polyP of ZFHX4. The inactive histone-lysine N-methyltransferase 2E (named MLL5, KMT2E) uses the C-terminal part composed of 3 large proline-rich fragments to interact with natural cytotoxicity receptors of the NKp44 natural killer cells [53]. We could not identify any specific function(s) for the translation initiator factor IF-2. In the case of the N terminus containing the proline-rich region of testis-specific glyceraldehyde-3-phosphate dehydrogenase (GAPDHS), it was shown to bind to the tail sperm cytoskeleton [54–56] and to stabilize the structure of the enzyme itself [57].

#### HHblits

Table 3 presents selected cases from the HHblits analysis. In each example, the query sequence (chloroplast sensor kinase, chloroplastic) is the same and it is a poly-S sequence without mutations. All examples have the same midline length, but none of them is perfectly matched to the query. However, they have lower e-value in comparison to perfectly matched alignments from BLAST of the same length.

**Table 3:** HHblits found more distant similarities. We selected alignment results where one of the sequence is from chloroplast sensor kinase, chloroplastic protein. The midline, ‘—’ mark indicates score above 1.5, while ‘+’ indicates score between 0.5 and 1.5

#	Protein name	Alignments	e-value
1	Chloroplast sensor kinase, chloroplastic (F4HVG8)	SSSSSSSSSSS (39 - 49)	1.6e-15
	midline	||||+||||||	
	Lamellipodin (Q70E73)	SLSSSSIKSGSSSSS (527 - 541)	
2	Chloroplast sensor kinase, chloroplastic (F4HVG8)	SSSSSSSSSSS (39 - 49)	3.2e-13
	midline	|||||||||||	
	E3 ubiquitin-protein ligase UBR4 (Q5T4S7)	AALAASSGSSSASSSSAPVAASS (3333 - 3355)	
3	Chloroplast sensor kinase, chloroplastic (F4HVG8)	SSSSSSSSSSS (39 - 49)	5.9e-15
	midline	||||+||||||	
	Dual specificity tyrosine-phosphorylation-regulated kinase 1B (Q9Y463)	SSSTASSISSSGGSSGSSS (459 - 477)	
4	Chloroplast sensor kinase, chloroplastic (F4HVG8)	SSSSSSSSSSS (39 - 49)	1.4e-14
	midline	|||||||||||	
	Nucleolar and coiled-body phosphoprotein 1 (Q14978)	DSSSDSDSSSSEEEEE (467 - 482)	
5	Chloroplast sensor kinase, chloroplastic (F4HVG8)	SSSSSSSSSSS (39 - 49)	9.6e-13
	midline	|||||+|||||	
	Krueppel-like factor 16 (Q9BXK1)	PGGASPASSSSAASSPSSG (94 - 112)	

For HHblits, alignments with a lower e-value are less similar than alignments with higher e-value found by BLAST, even if the input protein dataset is the same in both cases. Perfect alignment of serine of the same length in case of BLAST has an e-value equal to $2.95581e-05$ which is a huge difference in comparison to HHblits where the highest e-value from imperfect match is $3.2e-13$ (Table 3). Additionally, HHblits was not able to find perfect matches for a given region.

The first alignment has a better score and e-value than the second. Both of them have 100% similarity. Additionally, the second alignment has 2 mismatches and worse e-value than the first one with 3 mismatches. The third, the fourth and the fifth alignments have three mismatches and are assigned slightly different e-value, score and similarity. The third and the fifth hit sequences are 19 amino acids in length. Furthermore, the biggest difference among e-values is between the third and the fifth alignments. The fourth example is in the middle but has a different hit sequence length (16 aa).

**Figure 6:**
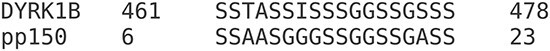
Protein low complexity region alignment of the human dual specificity tyrosine-phosphorylation-regulated kinase 1B (DYRK1B) and the small capsomere-interacting protein (pp150, pUL32, m48.2) of the cytomegalovirus obtained using FFAS03 algorithm [62].

For most of the LCRs from Table 3, we were not able to assign functions based on the literature search. However, we found an interesting similarity of the dual specificity tyrosine-phosphorylation-regulated kinase 1B (DYRK1B) to the small capsomere-interacting protein (pp150, pUL32, m48.2) of the cytomegalovirus (Figure 6). Pp150 is known to bind to capsid proteins, especially to Tri1, Tri2A and Tri2B [58, 59]. Experiments suggest that tegument protein pp150 contributes to a netlike layer that may stabilize the HCMV capsid [60]. LCR from Chloroplast sensor kinase, chloroplastic (F4HVG8) is located in the region of the transit peptide and it has to be rich in serine according to von Heijne *et al.* [61]. This serine homopolymer significantly enriches this region in the required type of amino acid which may be crucial to gain its function. We do not know the function of the LCR located in the Krueppel-like factor 16 (Q9BXK1). By similarity to the sensor kinase LCR and close vicinity to 3 zinc fingers, we speculate that it may interact with different proteins as a hinge structure.

Another example of similarity to the same serine-rich fragment of the chloroplast sensor kinase is the nucleolar and coiled-body phosphoprotein 1 (Nopp1, NOLC1) which acts as a platform to connect RNA polymerase I with enzymes responsible for ribosomal processing and modification [63]. Experiments suggest that this serine-rich fragment may be important in binding the TFIIB transcription factor [64].

#### CD-HIT

Our analysis of LCR results from CD-HIT is slightly different in comparison to BLAST and HHblits as results of CD-HIT are in the form of sequence clusters. Since the number of the sequences returned by CD-HIT was significantly smaller in comparison to BLAST and HHblits (Figure 4), we decided not to remove pairs in which proteins come from the same family. We noticed several interesting facts while analysing clusters created by the CD-HIT method.

In the Table 4, cluster 1 sequence P14922 is the representative of the cluster and consists of a tandem repeat of glutamine and alanine followed by a homopolymer of glutamine. This representative sequence joins two different types of LCRs to the cluster: homopolymers of glutamine and short tandem repeats of glutamine and alanine. As a result, we have sequences that are similar to representatives but it does not mean that other sequences in clusters are similar to each other. Next disadvantage of this approach is that homopolymers of glutamine (Q0CQ46) join the cluster where the representative sequence (P14922) is a combination of two types of LCRs. On the other hand, some of homopolymers of glutamine join clusters where the representative is also a homopolymer of glutamine. In such a case, we have wrong situation where homopolymers of glutamine are spreaded among different clusters.

**Table 4:** CD-HIT places dissimilar LCRs to the same clusters and similar LCRs to different clusters. Sequences where ^*^ is added to Uniprot AC are representatives of clusters

#	Example sequences in cluster	Uniprot AC	Number of LCRs in cluster
1	QQQQQQQQAQQQ	Q0CQ46	8 (99 cluster)
	QAQAQAQAQAQAQAQAQ	Q5ABZ2	
	QLQAQAQAQAQAQAQAQAQAQAQAQAQAQAQAQAQAQAQAQAQAQAHAQ AQAQAQAQAQAQQQQQQQQQQQQQQQQQQQQQQQQQQQQQQQLQPLPRQ QLQQ	P14922^*^	
2	GAGGGGGGGGGGGGSGGGGGGGGAGGAGGAAAAAAGAGAVAAQAQAQAA AAAAAAAAAAAGGGGGGGYGSSSSGYGV	E9Q4N7^*^	201(301 cluster)
	AAAAAAAAAAAAAAA	P35453	
	GGGGGGGGGTGGGGGGG	O77215	
3	QQQQQQQEQKQQLQQQQQQQQQLQQQQQQQQQQ	P04725	3 (233 cluster)
	LQQLQQQQQLQQQQQLQQQQQQQLQQQQQLQQQQLQQQQQQQQLQQQQQ QQLQQQQQQLQQQQQQQQQQFQQQQQQQQ	O14686^*^	
	LQRQRQQQQLQQQQQQQLQQQQ	P52288	
4	LQQQLSQQQQQLSQQQQQQQQLSQQQQQQLSQQQQQQLSQQQQQQLSQQ QQQQ	Q9ZTX8^*^	1 (743 cluster)
5	SSSSSSSSSSSSSSSSSSSSSSSSSSSSSSSSSSSSSSSSSS	Q8BTI8	119 (11 cluster)
	SSSSSSSSSSSSSSSSSSSSSSSSSSSSSSSSSSSSSSSSSSSSSSSSS SSSSSSSSSSSSSSSSSSSSSSSSSSSSSSSSSSSSSSSSSSSSSSSSS SSSSSSSSSSSSSSSSSSSSSSSSSSSSSSSSSSSSSSSSSSSSSSSSS SSSSSSSSSSSSSSSSSSSSSSSSSSSSSSSSSSSSSSSSSSSSSSSSS SSSSSSSSSSSSSSSSSSSSSSSSSSSSSSSSSSSSSSSSSSSSSSSSS SSSSSSSSSSSSSSSSSSSSSSSSSSSSSSSSSSSSSSSSSSSSSSSSS SSSSSSSSSSSS	Q75JC9^*^	
	SSSSSSSSSSS	P78424	
6	QQQVQAAAAAAAVAQAQAQAQAQAQAQAQAQAQAQASQASQQQQQQQQQ QQQQQQQ	Q8TF68^*^	1 (625 cluster)
7	QQQVQAAAAAAAVAQAQAQAQAQAQAQAQAQAQAQAQAQAQASQASQQQ QQQQ	Q9EQJ4^*^	1 (723 cluster)

Another disadvantage is that CD-HIT creates two different clusters from two highly similar sequences from composition and length perspectives. Table 4 contains an example of this situation. Sequence with Uniprot AC Q8TF68 which belongs to cluster 6 and sequence Q9EQJ4 which belongs to cluster 7 are highly similar but were assigned to different clusters as representative sequences. On the other hand, cluster 4 consists of the orphan sequence (Q9ZTX8). However, we can notice that this sequence is similar to the representative sequence in cluster 3. These results are correct because these sequences have different repetitive patterns. The sequence in cluster 4 is composed of the LSQQQQQQ motif, while the representative sequence in cluster 3 is composed of the LQQQQQ motif.

The fifth cluster from Table 4 (5th row) consists of serine homopolymers of different lengths. LCR from protein Q75JC9 has the longest sequence with 306 serines. The same cluster contains a region from P78424 with 11 serines. Such differences in length can be reduced by changing the cutoff option of CD-HIT, which may be adjusted by the user for a specific problem. Higher values of the cutoff option results in lower diversity of sequence lengths in clusters. By default, the parameter is turned off. Detailed analysis of this parameter is provided in Supplementary material.

## Discussion and conclusions

Our analysis confirms that selected methods are most efficient for comparing high complexity protein sequences as they rely on statistical models designed for sequences with diverse amino acid composition. This is why masking low complexity parts improves searching for homologous proteins [25]. An obvious way to include LCRs into the analysis is to disable the masking. This still does not solve the problem related to the fact that these methods were optimized for HCPs of sequences.

The methods analysed in this paper use general purpose scoring matrices. They are efficient to align typical protein sequences but fail while applying them for non-standard amino acid composition of sequences such as LCRs [65]. For example, BLOSUM, one of the most popular scoring matrix, was built from the BLOCK database which contained only about a promile of proteins with LCRs [66]. Sequence regions in proteins with non-standard amino acid composition (especially LCRs) have their own structural and amino acid preferences [1, 65]. For example, A-rich and L-rich regions promote alpha-helix formation, while H-rich and P-rich regions frequently overlap with disorder regions [67]. Whenever possible, it is recommended to use specialised scoring matrices for different types of protein domains such as intrinsically disordered regions [68]. Unfortunately, many tools for protein sequence comparison lack of parameter which enable scoring matrix selection or they allow to select predefined set of matrices only.

MSA and consequently a dataset of profiles of Hidden Markov Models for LCRs may be created in many different ways. One problematic case is shown in Figure 3 where three selected tools gave three different results. Only one method was able to align the valine correctly. The problem presented is well known as shift-errors (the erroneous positioning of a single indel whose length is preserved) and occurs in protein and genome sequences [69]. Such kind of error is especially abundant in LCR alignments. To solve this kind of error, we need data about evolution of proteins, but in many cases, it is limited due to lack of ancestral sequences [70]. MSA creation is a general issue related to HCRs as well. It is well known that automatically generated MSA need to be manually curated [71, 72].

E-value is a statistic useful while searching for similarities that have biological meaning as it provide the estimate if a particular similar sequence may occur in a database by a chance [73]. However, in the case of LCRs, especially homopolymers, the number of even identical sequences in a given database may vary a lot because in nature, some of homopolymers occur more frequently than others [74]. Therefore, to assess the significance of an alignment of LCRs, we suggest to pay more attention to score, identity and similarity metrics than e-value.

Local alignment causes loss of information about length of longer LCRs than query sequence. Such a case is presented in Table 2 for homopolymers of proline (alignment 5). BLAST compares entire poly-P region from *Zinc finger homeobox protein 4* protein to only a part of poly-P region from *Glyceraldehyde-3-phosphate dehydrogenase,testis-specific* protein. Therefore, local alignment based tools lose information about difference in LCR lengths. However, current knowledge says that homopolymer length may affect protein function. For instance, poly-Q region may cause neurological disease if it reach a given length [75]. Therefore, differences in lengths should be somehow penalized in LCR alignments for example by comparing them globally. Another approach to analyse the similarity of LCRs found in the literature is a metric based on Jaccard index between seqeunces [76]. In this solution, the Jaccard score may be easily penalised by difference in the number of residues between composition vectors.

Selection of representative sequences in CD-HIT may result in wrong assignment of sequences of different types of LCRs. This is especially visible in LCRs that consist of two different types of LCRs. As mentioned in Results section, if a representative sequence consists of two types of LCRs, then three types of LCRs may join this cluster: LCRs of the first type, LCRs of the second type and the combination of both LCRs. As a result, a single cluster contains different types of LCRs. Such a scenario is present in examples 1 and 2 in Table 4. The problem occurs because CD-HIT uses local alignment to join sequences to cluster that are similar to representative. A metric designed for LCR comparison is crucial for better performance of general purpose protein sequence clustering since many methods use it to create clusters [77, 78]. Another interesting approach to cluster LCRs is through tagging them by their types [79].

Even if we showed that standard methods are not appropriate for the similarity analysis of LCRs we could identify some examples that are statistically sound (see Results section for BLAST and HHblits). In this work, we collected a few examples of LCR similarity/identity that may follow the rule of transitivity. If the function of one LCR is known and we show its similarity to another LCR, then we may hypothesize that the role of the second LCR may be identical or similar, e.g. if LCR1 binds a protein, we may argue for an analogous binding mechanism of a similar LCR2.

Our work focuses on three state-of-the-art methods for protein sequence comparison that are based on the most popular approaches for protein similarity analysis such as local alignment or profiles of Hidden Markov Models. There are other tools for protein sequence comparison that can be found in the literature, but they usually rely on similar approaches. In this paper, we point out why these methods are not suitable for LCR analysis. However, since there are no tools specifically designed for LCRs, we suggest to use BLAST. It seems to be the best choice to search for similar LCRs. HHblits may also be useful in LCR analysis, but for more distant similarities. Finally, we conclude that CD-HIT cannot be used for analysing LCRs as the local comparison to representative sequences results in badly clustered sequences that are not similar to each other. Scientists should be aware of these drawbacks while using these methods for searching for similar LCRs. On the other hand, our results may be used to improve existing methods or to design new ones especially crafted for LCR comparison.

**Key Points**

- We would like to alert the community that similarity searches reported by canonical tools may result with false positive hits, even if they use the optimal parameter set for these methods.
- We indicate which design assumptions of the selected methods are not suitable for the analysis of low complexity regions (LCRs).
- In the article, we advise on how to adjust HHblits and CD-HIT parameters to find similar LCRs more efficiently.
- This knowledge can be used to improve existing methods or to create new methods specifically designed to analyze the similarity between low complexity regions.

## Supplementary Material

canonical_methods_supplementary_bbac299

## Data Availability

The data underlying this article are available in the UniProtKB/Swiss-Prot at www.uniprot.org, and can be accessed with major release-2020_04.

## Author Biographies

**Patryk Jarnot** is a Research Assistant in the Department of Computer Networks and Systems, Silesian University of Technology. He develops methods for the analysis of protein domains with non-standard amino acid composition.

**Joanna Ziemska-Legiecka** is a PhD student at the Institute of Biochemistry and Biophysics PAS, where she studies low complexity regions of proteins.

**Marcin Grynberg** is an Associate Professor at the Institute of Biochemistry and Biophysics PAS, where he studies low complexity regions of proteins.

**Aleksandra Gruca** is an Associate Professor in the Department of Computer Networks and Systems, Silesian University of Technology. Her research focus on machine learning methods for protein sequence data analysis and application of predictive models for the integrated analysis of large-scale heterogeneous datasets.
