# Supplementary material for: Insights from analyses of low complexity regions with canonical methods for protein sequence comparison
Source: Brief Bioinform. 2022 Aug 1;23(5):bbac299. doi: 10.1093/bib/bbac299 (PMC9487646; doi:10.1093/bib/bbac299)
Supplement: canonical_methods_supplementary_bbac299 [file canonical_methods_supplementary_bbac299.pdf]

Comparison of statistical analysis for low and high complexity regions

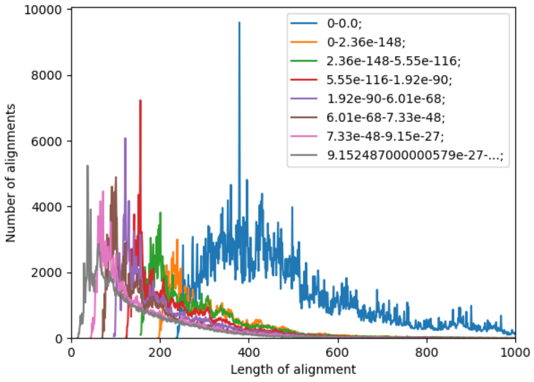

a) BLAST: length of HCR alignments to number of HCR alignments

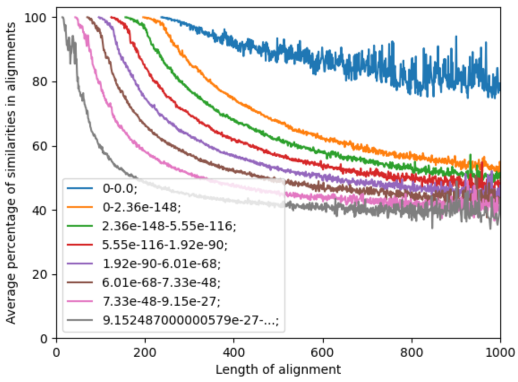

b) BLAST: length of HCR alignments to average similarity of sequences in HCR alignments

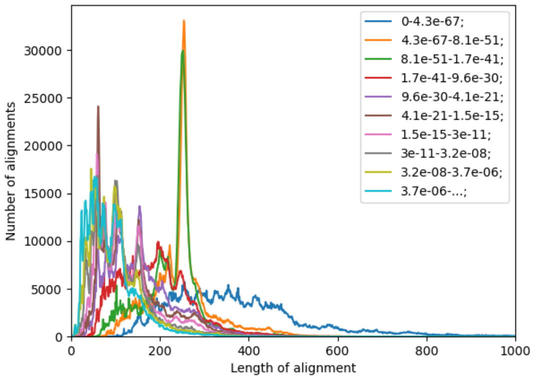

c) HHblits: length of HCR alignments to number of HCR alignments

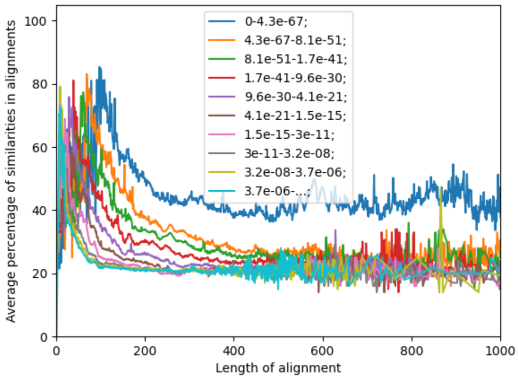

d) HHblits: length of HCR alignments to average similarity of sequences in HCR alignments

Figure S1. HCR results differ from LCR results. For both BLAST and HHblits results, e-value depends on length and similarity. We sorted results by e-value and divided them into 10 groups of similar size. Figures S1.a and S1.c show the distribution of the alignments by length. Figures S1.b and S1.d illustrate how the alignments of a given length are on average similar.

Figure S1 corresponds to Figure 4 that shows the same diagrams for LCRs. Figure S1.a shows that each percentile has an alignment length which is dominant in terms of the number of alignments (demonstrated by different peaks in number of alignments for each percentile). In contrast to Figure 4.a these dominant values are separated on the x axis. This is because LCRs are mostly short sequences (Jarnot et al. 2020) and the difference between percentiles decreases along with e-value. Similar scenarios can be observed for

Figure S1.b and Figure 4.b. For LCRs, the first percentile contains all long and well matched alignments. Length of alignments in case of HCRs is distributed more linearly since the length variation in HCRs is larger.

From Figure 4.c we can conclude that in case of LCRs e-value is not relevant to length of alignment. Each percentile contains a similar number of alignments of the same length. However, from Figure S1.c we can read that for HCRs along with e-value of percentiles, the length of alignments increase. In Figures 4.d and S1.d we can see that difference in similarity of alignments is visible among percentiles for both LCRs and HCRs.

Methods' parameters adjustments

To find the best parameters for each method, we read each method's guidelines and checked the different parameter sets. For BLAST please read an article describing the LCR-BLAST which proposes a set of parameters for LCRs (Jarnot et al. 2020). For HHblits and CD-HIT parameters please see the corresponding sections below.

HHblits

In this subsection we provide analysis of different HHblits parameters which are presented in Table S1. We analyse them by removing a single parameter from the optimal parameter set, showing the number of similar pairs after removal and discussing selected examples in detail.

#	Parameters	Missing parameter in comparison to #1	Number of similar pairs
1*	-id 100 -diff 0 -norealign -sc 0 -noprefilt		4 331 254
2	-diff 0 -norealign -sc 0 -noprefilt	-id 100	4 357 676
3	-id 100 -norealign -sc 0 -noprefilt	-diff 0	4 354 196
4	-id 100 -diff 0 -sc 0 -noprefilt	-norealign	623 205
5	-id 100 -diff 0 -norealign -noprefilt	-sc 0	4 002
6	-id 100 -diff 0 -norealign -sc 0	-noprefilt	2 309 843

Table S1. Number of similar pairs for different parameters for HHblits method in comparison to proposed set of parameters. The first row shows results for the parameter set used for LCR analysis in the main article. Each next row shows results for the parameter set where one is missing in comparison to the first row.

Parameters: -id 100 and -diff 0

These parameters are provided to increase similarity between sequences. If we add either *id* or *diff* parameters, the number of similar pairs decreases. On the other hand, detected

similar sequences are less distant (more similar). Figure S2 presents example alignments which represent removed pairs of sequences from results by adding *id* and *diff* parameters. Improvement is only compositional, therefore results still contain wrong hits described in the main article.

```
NNNNNNNNNNNSNNNSS
+|+.+|+++++|+|.||
SSSGGSGNSSGSSSRSS
```

a) *id* parameter missing

```
EELEEEEELEEEEEEELGED
.|+|+.||+|++|++++.||
KEKEKKKDKEKKEKKRKRED
```

b) *diff* parameter missing

Figure S2. Alignments found in results without either *id* or *diff* parameters. Figure S2.a shows the case where the homopolimer of Asparagine is considered similar to the Serine-rich region. Figure S2.b compares a negatively charged sequence to a positively charged.

Parameters: -norealign, -sc 0 and -noprefilt

Each of the pointed parameters increases the number of similar pairs. Even if sequences are composed of different amino acids they still share similar properties. Such a case is presented in Figure S3.a where the first sequence is biased towards Aspartic Acid while the second one is biased towards Glutamic Acid. However, both residues are negatively charged and acidic. Other two examples presented in Figure S3.b and S3.c share compositional similarity.

```
ELEDDRDDDDDDD
||||++||||+||
EEEEEEEDEEKDD
```

a) *norealign* parameter added

```
SSSSSSLSSSSSNSM
||||||+||||||||
SLSSSSIKSGSSSSS
```

b) *sc* parameter added

```
AAVAAAPVAADAAPAA
|+||||||.|||||+|
AEKAKAAALAAAAADA
```

c) *noprefilt* parameter added

Figure S3. Alignments found in results with *norealign, sc* and *noprefilt* parameters. Alignment presented in Figure S3.a compares sequences with similar properties found in results generated with the *norealign* parameter. Figures S3.b and S3.c show results generated with *sc* and *noprefilt* parameters respectively where both of them compare sequences of similar composition.

CD-HIT

#	Parameters	Number of similar pairs
1*	-l 4	237 782
2	default	206 367
3	-l 4 -s 0.7	32 039

Table S2. Number of detected similar sequences for different parameter sets by CD-HIT method. Results from the first row were used in the main article. The second row shows how

many sequences were in default results. The third row shows the number of sequences with limited difference in length between sequences to 70%.

Parameter: -l 4

This parameter is responsible for filtering out sequences which are shorter or equal to its value. LCRs are frequently short sequences therefore we recommend setting this parameter to its minimal value. The shortest sequence in our dataset is 9 residues long. However, the value 4 has been selected since it is the lowest allowed value and CD-HIT lacks the possibility to switch it off. As a result, we increased the number of similar pairs by over 31 thousand.

Parameters: -l 4 -s 0.7

Cluster	Sequence	UniprotACC
1	QQQQQQQQQQQQQQQQQQQQQQQQQQQQQQQQQQQQQQ	Q559R1
	QQQQQQQQQQQQQQQQQQQQQQQQQQQQQQQQQQQQQQQ	Q498D1
	QAQQQQQQQQQQQQQQQQQQQQQQQQQQQQQQQQQQQQQQ<br>QQQQQPQQPAPTQQ	Q4P209
	QQQQQQQQQQQQQQQQQQQQQQQQQQQQQQQQQQQQQQ	Q54NI1
	QQQQQQQQQQQQQQQQQQQQQQQQQQQQQQQQQQQQQQQ	P0CB66
	HQQQQHQQQQQQQQQQQQQQQQQQQQQQQQQQQQQQQQ	Q551F8
2	QQKQQQQQQQQQQQQQQQQQQQQQQQQHQQQQQHQQQQQH<br>QQQQQHQQQQQQ	Q6CPW4
	QQQQQQQQQQQQQQQQQQQQQQQQQQQQQQQQQQQQQ	P58462
	QQQQQQQQQQQQQQQQQQQQQQQQQQQQQQQQQQQQQ	Q55FT4

Table S3. Two example clusters found using -l 4 -s 0.7 parameter set. All homopolymers are almost identical, but some of them belong to different clusters.

The parameter determines the maximal difference in length between two comparing sequences. To show how it works in example we set its value to 70%. It has positive and negative impacts on results. Clusters contain less sequences which are not similar to each other due to alignment of incoming sequence to subLCR of the representative. On the other hand, the parameter decreases the number of similar pairs and some of similar sequences are assigned to different clusters. This is caused by the hard threshold provided by the introduced parameter. Table S3 presents two clusters which contain highly similar sequences. Protein Q559R1 is highly similar to protein Q55FT4, they differ just in a single residue, but these proteins belong to different clusters. Additionally, CD-HIT appends each sequence only to one cluster therefore subLCRs are still badly clustered.

Bibliography

Jarnot, Patryk, Joanna Ziemska-Legięcka, Marcin Grynberg, and Aleksandra Gruca. 2020. “LCR-BLAST—A New Modification of BLAST to Search for Similar Low Complexity Regions in Protein Sequences.” *Advances in Intelligent Systems and Computing*. https://doi.org/10.1007/978-3-030-31964-9_16.
